# Quantitative evaluation of recall and precision of CAT Crawler, a search engine specialized on retrieval of Critically Appraised Topics

**DOI:** 10.1186/1472-6947-4-21

**Published:** 2004-12-10

**Authors:** Peng Dong, Ling Ling Wong, Sarah Ng, Marie Loh, Adrian Mondry

**Affiliations:** 1 Medical and Clinical Informatics Group, Bioinformatics Institute, BMRC, A*STAR, Singapore

## Abstract

**Background:**

Critically Appraised Topics (CATs) are a useful tool that helps physicians to make clinical decisions as the healthcare moves towards the practice of Evidence-Based Medicine (EBM). The fast growing World Wide Web has provided a place for physicians to share their appraised topics online, but an increasing amount of time is needed to find a particular topic within such a rich repository.

**Methods:**

A web-based application, namely the CAT Crawler, was developed by Singapore's Bioinformatics Institute to allow physicians to adequately access available appraised topics on the Internet. A meta-search engine, as the core component of the application, finds relevant topics following keyword input. The primary objective of the work presented here is to evaluate the quantity and quality of search results obtained from the meta-search engine of the CAT Crawler by comparing them with those obtained from two individual CAT search engines. From the CAT libraries at these two sites, all possible keywords were extracted using a keyword extractor. Of those common to both libraries, ten were randomly chosen for evaluation. All ten were submitted to the two search engines individually, and through the meta-search engine of the CAT Crawler. Search results were evaluated for relevance both by medical amateurs and professionals, and the respective recall and precision were calculated.

**Results:**

While achieving an identical recall, the meta-search engine showed a precision of 77.26% (±14.45) compared to the individual search engines' 52.65% (±12.0) (p < 0.001).

**Conclusion:**

The results demonstrate the validity of the CAT Crawler meta-search engine approach. The improved precision due to inherent filters underlines the practical usefulness of this tool for clinicians.

## Background

Healthcare has been steadily moving towards Evidence-Based Medicine (EBM) since the term was formally introduced in 1992 by a group led by Gordon Guyatt at McMaster University, Canada [1-3]. EBM promotes systematic literature review, critical appraisal skills and integrates scientific evidence with clinical expertise in the daily management of patients. The first three steps involved in the practice of EBM can comprehensively be summarized as a one-page written paper on a particular clinical topic, which is most commonly called a 'Critically Appraised Topic' (CAT) [4]. Different acronyms have emerged in various specialties, such as Best Evidence Topics (BET) [5] in emergency medicine and Evidence-Based Journal Club Reviews (EBJCR) [6] in pediatric critical care medicine. All these essentially provide physicians with a systematic method of formulating a clinical question and then critically evaluating the literature to answer the question posed.

With the use of resources on the World Wide Web becoming common practice, several academic and healthcare organizations have built online CAT libraries for knowledge sharing with peer physicians. The repository of CATs has been growing steadily since the setup of the first accessible CATBank developed by the Centre for Evidence Based Medicine, Oxford in 1992 [7]. Among those, BestBETs developed by the Emergency Department, Manchester Royal Infirmary [8] and UMHS by the Department of Pediatric, University of Michigan Health System, Ann Arbor [9] hold hundreds of distinct topics. They are furnished with individual search engines for fast and direct access to a particular topic. Given the wealth of such medical information scattered in cyberspace, the effectiveness of locating the correct information has become an important issue [10].

### The CAT Crawler application

It is believed that more CATs will be added into the repositories as more people participate in EBM practice. However, the non-standardized electronic format of CATs has created much difficulty for physicians to access a particular topic. Accordingly, the CAT Crawler was developed at the Bioinformatics Institute, Singapore [11,12] to provide a one-stop search and download site for physicians by setting up a common platform to access eight popular online CAT libraries. CAT Crawler is freely accessible online [12].

The core component of the CAT Crawler is a meta-search engine. Its search is currently based on CAT resources from eight public online libraries [11]. Once the user chooses the libraries he intends to use in the search, information tailored to his needs can be produced. The matched results are sorted according to their origins.

Following the user input of a query keyword, a partial search is done through information extracted during an off-line process from six websites that do not hold search engines.

The remaining search is carried out by querying the two individual search engines at BestBETs and UMHS. Use of the CAT Crawler is expected to have a quantitative and qualitative improvement of the retrieved results by post-processing obtained raw results from both libraries.

### Motivation of the evaluation

The work presented here aims to evaluate the quantity and quality of the obtained results from the CAT Crawler meta-search engine, and thus to evaluate the validity and the usefulness of the application. Recall and precision were estimated to measure the performance of this meta-search engine versus the two individual search engines at BestBETs and UMHS.

## Methods

**Figure 1:**
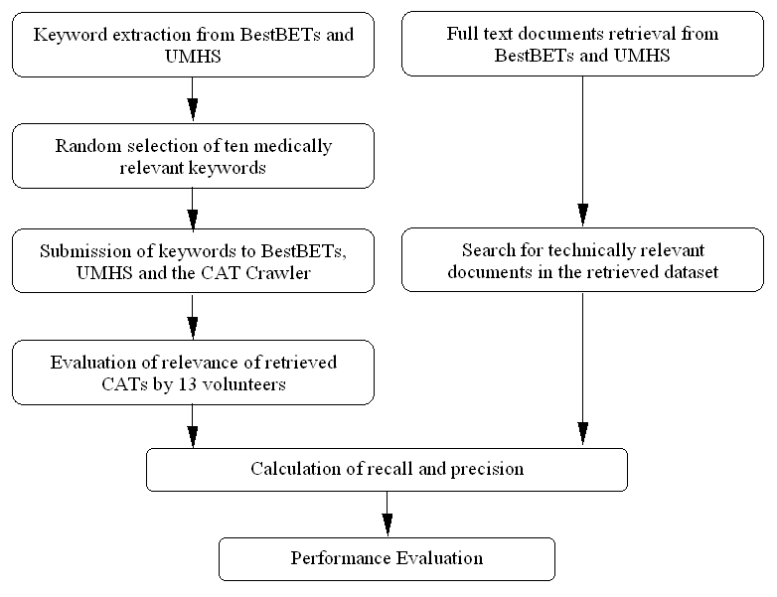
Workflow for evaluation of the CAT Crawler meta-search engine

The workflow of this study is demonstrated in Figure 1.

### Selection of ten query keywords

To find a viable sample of keywords for a test search, the titles of all CATs stored in the two CAT libraries, namely BestBETs and UMHS were submitted to *AnalogX Keyword Extractor*, which is freely available online [13]. This led to a list of around 2000 keywords, of which approximately 500 were present in both libraries, of which ten were randomly chosen. In a second step, that list was curated so that only medically relevant keywords remained, excluding words such as *and *and *day*.

### Search for technically relevant documents in the dataset

In order to be able to calculate recall as detailed below, the *technical relevance *of all documents in the dataset must be assessed. In this study, a document is called technically relevant for a given search term if it contains this term in the full-text. *Perl *scripts were developed to examine all CATs in the two libraries BestBETs and UMHS and the total number of relevant documents as per the above definition in each library was collected for further calculation. This was done for each selected keyword and the process was independent from the search using the three search engines: the CAT Crawler, BestBETs and UMHS.

### Relevance evaluation of the retrieval results

In the next step, those ten keywords were submitted to the search engines at BestBETs and UMHS, and to the CAT Crawler meta-search engine. The retrieved links were evaluated for their relevance by 13 volunteers, who are categorized into three groups. Among them, one physician in Group I represents medical professionals, six persons in Group II represent people who were trained in biology or medicine, and six persons in Group III represent people who do not have any medical background.

### Calculation of recall and precision

Recall and precision are two accepted measurements to determine the utility of an information retrieval system or search strategy [14]. They are defined as:


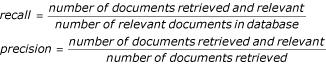


Despite the relevance evaluation from 13 volunteers, it is necessary to know the total number of the relevant documents in a database for each query keyword in order to estimate the recall. In the present study, a particular CAT in a database was defined as technically relevant if the keyword could be found in its full-text article.

The CAT Crawler is designed not to hold permanently any full-text CATs [11]. When a query is done choosing the option to search only BestBETs and UMHS, the total number of relevant document in its acute database is equivalent to the sum of the number of relevant documents in the two libraries BestBETs and UMHS. Accordingly, the recall and precision of the CAT Crawler meta-search engine are revised as:


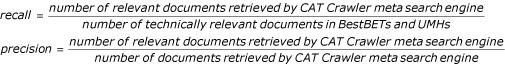


Similarly, the recall and precision of the search engines at BestBETs and UMHS are estimated based on the combined repository of the two individual sites. The revised formula are shown below:


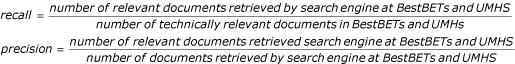


### Performance evaluation of the CAT Crawler versus BestBETs and UMHS

The averaged precision and recall over all evaluators are used to evaluate the performance of the CAT Crawler meta-search engine. These values are compared to the estimate based on the search results from the two individual search engines at BestBETs and UMHS.

## Results

### Ten keywords for the search engine evaluation

According to the predefined selection criteria, the ten keywords listed in Table 1 were selected as the seed for a test search. The number of retrieved results from each search engine was gathered with respect to each keyword query. For the selected ten medically relevant keywords, the total number of matched results are 116, 65 and132 corresponding to the three search engines at BestBETs, UMHS and CAT Crawler. The difference of 49 retrievals between the CAT Crawler and the sum of BestBETs and UMHS reflects the meta-search engine's inherent filter function which is described previously [11].

### Performance evaluation of the CAT Crawler versus BestBETs and UMHS

**Figure 2:**
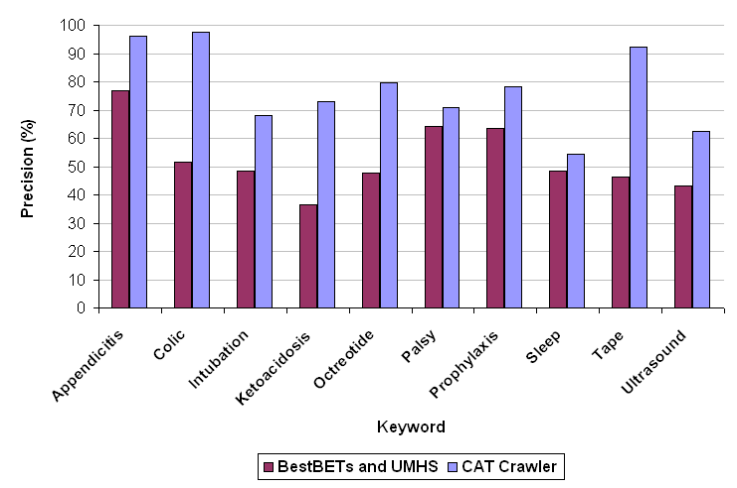
Precision plot of the CAT Crawler meta-search engine and two individual search engines

To compare the performance of the CAT Crawler meta-search engine to that of the two individual search engines, recall and precision were computed and averaged over the evaluation of all 13 participators. The data recorded are shown in Table 2. As the CAT Crawler meta-search engine is built upon the two individual search engines, the document collection for evaluation is the combined repository of BestBETs and UMHS. The retrieved relevant documents from the CAT Crawler are the same as that from the individual search engines. This leads to the identical recall for both cases (Table 2). The average precision is increased from the individual search engines' 52.65% (±12.0) to the CAT Crawler's 77.26% (±14.45). Figure 2 provides a more intuitive comparison corresponding to each keyword.

## Discussion

The performance evaluation clearly places the CAT Crawler meta-search engine on par with the individual search engines at BestBETs and UMHS as far as recall is concerned, and well above them for precision (see Table 2 and Figure 2). According to these results, the application can be called successful: by using the CAT Crawler to look for relevant information at specific sites, the medical professional will obtain as much information as by going to the sites directly, but the precision of the obtained results will be higher.

Benoit [15] has analyzed various methods of information retrieval and their impact on user behavior. He finds that users wish for greater interactive opportunities to determine for themselves the potential relevance of documents, and that a parts-of-document approach is preferable for many information retrieval situations. At present, the CAT Crawler allows a number of interactive opportunities [11], but their implementation would have no impact on the calculation of recall and precision under the condition of the present study. Benoit's reasoning should be kept in mind, however, for improving the user friendliness in the sense that some further useful filter functions can be included in future versions of the application. While such advanced search functions will be profitable when large datasets are studied, the currently still manageable information in the online CAT libraries [11] will serve the user better if initially displayed in a broader way. For example, some of the information displayed here may be older than 18 months, which makes it undesirable according to the strict rules for CAT updating as defined by Sackett et al [3]. Formally outdated information, however, may in a given situation still be "best evidence" and positively influence the decision-making. Use of filters to block aged information will certainly influence this process.

Despite the encouraging results, some fundamental questions regarding the evaluation of this meta-search engine in particular, and also meta-search engines in general remain unsolved.

With regard to recall, there is the theoretical possibility that manually searching all documents at a given repository will yield a higher recall for a given search term. In view of hundreds of CAT documents per repository, however, it seems unlikely that a human evaluator's attention will not wander, leading to less than optimal scrutiny of the documents and introducing a non-quantifiable error to the evaluation. This is a general problem of knowledge databases, especially when indexing is done by humans, whose decisions are not consistent. In a study of 700 Medline references indexed in duplicate, the consistency of main subject-heading indexing was only 68% and that for heading-subheading combinations was significantly less [16]. Also, in two studies [17,18] on Medline searching, there was considerable disagreement by those judging relevance of the retrieved documents regarding which documents were relevant to a given query.

In order to overcome this problem, the number of documents that contained a given keyword as found by the keyword extractor was used as the basis for calculating the technical recall. This may (or may not) lead to numerical results for recall that differ from the absolute true value as determined above. As the same numbers are used throughout, however, the comparison of search results obtained by the individual search engines and the CAT Crawler meta-search engine remains valid.

Critics have pointed out the over-reliance of researchers on the use of recall and precision in evaluation studies [18] and the difficulty to design an experiment that allows both laboratory-style control and operational realism [19]. For instance, recall may be of only little consequence once the user has found a useful document. Rhodes and Maes [20] evaluated both with a traditional field user test and then asked for relevance feedback. In their experiment, users gave a score 1–5 to each document that was delivered to calculate an overall average value for perceived precision. While a document can get a high score for precision, it may at the same time get a low score for practical usefulness. This was often due to the fact that the documents were already known to the users, in some cases had even been written by them. Accordingly, Rhodes and Maes [20] added features to the system that weeded out relevant documents that by some predefined criteria would not be useful. As a result, the measurable precision could be worse, but the overall usefulness could be better. In the study presented here, a similar approach was chosen in the instructions to the evaluators in the sense that they could make the distinction between 'irrelevant' (e.g. the retrieved document was only a web hosted clinical question) and 'medically irrelevant' (e.g. the word *Appendicitis *appeared only in the reference section of a document dealing with questions of abdominal pain relief). Due to the relatively small number, no difference could be detected between the various grades of relevance, and results were pooled to relevant/irrelevant and used for calculating recall and precision as described above. If a larger number of volunteers could be recruited, repetition of this evaluation might yield interesting results.

Other approaches have been spawned to evaluating system effectiveness in order to minimize these problems with recall and precision. One example are task-oriented methods that measure how well the user can perform certain tasks [21-24]. These different approaches were not chosen in this study for a reason: the primary aim was to compare the search engines. Under the present restrictions, recall and precision allow to answer this question.

## Conclusions

In summary, the data obtained from the analysis of search results obtained from identical queries submitted to the two CAT libraries at BestBETs and UMHS, using either their respective search engines or the CAT Crawler meta-search engine, showed a competitive recall, and superior precision of the meta-search engine compared to the individual search engines.

## Competing interests

The author(s) declare that they have no competing interests.

## Authors' contributions

PD participated in the design of the study, data analysis and drafting of the manuscript. LLW and SN generated raw data for the study. ML was involved in drafting the manuscript. AM designed the study and participated in the drafting of the manuscript.

## Pre-publication history

The pre-publication history for this paper can be accessed here:



## Figures and Tables

**Table 1:** Ten random keywords and corresponding number of retrieved results from search engine at BestBETs, UMHS and CAT Crawler

Keyword	Search Engine		
	
	BestBETs	UMHS	CAT Crawler
Appendicitis	7	3	8
Colic	15	2	9
Intubation	26	5	22
Ketoacidosis	2	2	2
Octreotide	3	2	3
Palsy	6	5	10
Prophylaxis	18	19	30
Sleep	5	13	16
Tape	4	2	3
Ultrasound	30	12	29
	116	65	132

**Table 2:** Numerical recall and precision for the CAT Crawler meta-search engine and two individual search engines at BestBETs and UMHS

	Recall (%)		Precision (%)		
	BestBETs & UMHS	CAT Crawler	BestBETs & UMHS	CAT Crawler	p-value

Appendicitis	96.15	96.15	76.92 (±4.80)	96.15 (±6.00)	0.000
Colic	54.81	54.81	51.58 (±2.58)	97.44 (±4.87)	0.000
Intubation	44.12	44.12	48.39 (±13.56)	68.18 (±19.10)	0.130
Ketoacidosis	48.72	48.72	36.54 (±12.97)	73.08 (±25.94)	0.001
Octreotide	59.62	59.62	47.69 (±10.13)	79.49 (±16.88)	0.000
Palsy	70.77	70.77	64.34 (±16.37)	70.77 (±18.01)	0.002
Prophylaxis	67.03	67.03	63.41 (±11.60)	78.21 (±14.31)	0.074
Sleep	57.95	57.95	48.29 (±19.82)	54.33 (±22.30)	0.038
Tape	46.15	46.15	46.15 (±7.31)	92.31 (±14.62)	0.000
Ultrasound	42.22	42.22	43.22 (±8.06)	62.60 (±11.68)	0.017
Average	58.75 (±16.25)	58.75 (±16.25)	52.65 (±12.0)	77.26 (±14.45)	0.000
